# Supplementary material for: Enhanced Heterogeneous Peroxymonosulfate Activation by MOF-Derived Magnetic Carbonaceous Nanocomposite for Phenol Degradation
Source: Materials (Basel). 2023 Apr 24;16(9):3325. doi: 10.3390/ma16093325 (PMC10179389; doi:10.3390/ma16093325)
Supplement: Supplementary file 1 [file materials-16-03325-s001.zip › materials-2359275-supplementary.pdf]

Supplementary Materials

Enhanced Heterogeneous Peroxymonosulfate Activation by MOF-Derived Magnetic Carbonaceous Nanocomposite for Phenol Degradation

Xinyu Li [1,2], Xinfeng Zhu [2], Junfeng Wu [2], Hongbin Gao [2], Weichun Yang [1,*] and Xiaoxian Hu [2,*]

[1] Chinese National Engineering Research Center for Control & Treatment of Heavy Metal Pollution, Institute of Environmental Engineering, School of Metallurgy and Environment, Central South University, Changsha 410083, China
[2] Henan Key Laboratory of Water Pollution Control and Rehabilitation, Henan University of Urban Construction, Pingdingshan 467000, China
* Correspondence: yang220@csu.edu.cn (W.Y.); 20221057@hncj.edu.cn (X.H.); Tel.: +86-138-7584-6982 (W.Y.); +86-152-9226-7733 (X.H.)

This document contains

1 Text

2 Figures

3 Tables

4 References

Text S1. Material characterization.

The elements relative contents and chemical states of the samples were measured by an X-ray photoelectron spectroscopy (XPS, Thermo K-Alpha+). X-ray diffraction (XRD) patterns were performed by Bruke D8 Advance at 40 kV and 40 mA (AXS, Germany). The N_2 adsorption isotherms were measured at 77 K by an ASAP 2460 (America). Brunauer-Emmett-Teller (BET) and Barrett-Joyner-Halenda (BJH) methods were used to analyse the specific surface area and pore size distribution respectively. Fourier transform infrared (FT-IR) spectra were recorded on a FT-IR spectrometer (Nicolet IS 10, Thermo Fisher Scientific Inc., America).

Text S2 Catalytic experiments

Briefly, the adsorption-resolution equilibrium was achieved by dosing an amount of catalyst into a solution of phenol at a concentration of 20 mg L^{-1} (50 mL) and stirred for 30 min. Then the catalytic reaction was initiated by adding a certain amount of PMS. At predetermined time intervals, 2 mL reaction solution was filtered and immediately injected into 0.5 mL of methanol for terminate further reaction. The pH of solution was adjusted by 0.1 M H_2SO_4 or NaOH. For the reusing tests, the catalysts were separated by centrifugation from the reaction solution after each run of the experiment, then washed thoroughly with plenty of deionized water. Four kinds of quenching reagents, i.e., methanol (MeOH), tert-butyl-alcohol (TBA), furfuryl alcohol (FFA), and parabenzoquinone(p-BQ) were used to determine the types of active radicals generated in the reaction.

The concentrations of phenol were detected by an ultra-performance liquid chromatography (UPLC, Waters, ACQUITY H-Class) with a BEH C18 column (1.7 μm, 50 × 2.1 mm, Waters). A mobile phase consisted of 30% acetonitrile (A) and 70% phosphate buffer (B) with a flow rate of 0.3 mL min^{-1}. The UV detector wavelength was set at 270 nm. The retention time was approximately 1 min.

The CV tests were conducted at room temperature. The reference electrode was Ag/AgCl, the counter electrode was Pt sheet, and the working electrode was GCE coated with the tested material. The tests were conducted in 0.5 M of

Na_2SO_4, 2 mmol L^{-1} of PMS and 20 mg L^{-1} of phenol mixed solution. The CV measurement range was -1.0 to 1.0 V, and the scanning rate was 10 mV s^{-1}.

Text S3 Pseudo-first-order kinetic model

The removal rate of phenol was fitted to a pseudo-first-order kinetic model as follows:

$$\ln C_t/C_0 = -k_{obs}t \quad (1)$$

where C_0 (mg L^{-1}) is the initial phenol concentration, C_t (mg L^{-1}) is the concentration of phenol at time t, and kobs (min^{-1}) is the first order rate constant of phenol degradation.

Text S4 Cost-benefit analysis

According to the experimental conditions in the manuscript, the cost of catalysts and oxidants for treating 1 mg of phenol is calculated as follows: the cost of catalysts is 0.0158 $/mg, and the cost of oxidants is 0.0043 $/mg. The costing estimated is based on laboratory prices for analytical reagents. In contrast, the cost of chemical reagents used in the large-scale production of catalysts are lower than laboratory cost estimates. The catalysts in this study have excellent recyclability and recoverability. Therefore, it can save the application cost of catalysts.

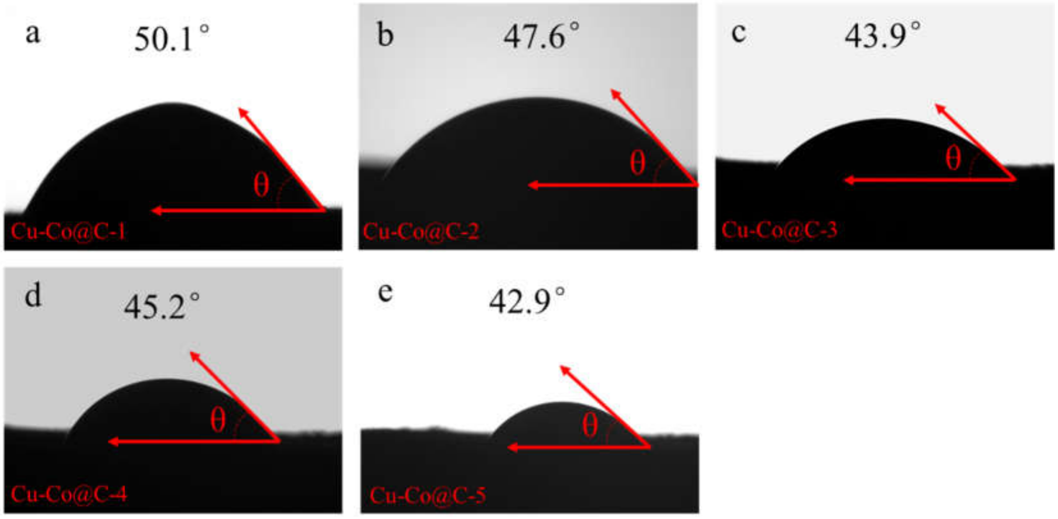

Figure S1. Water contact angles of Cu-Co@C-x.

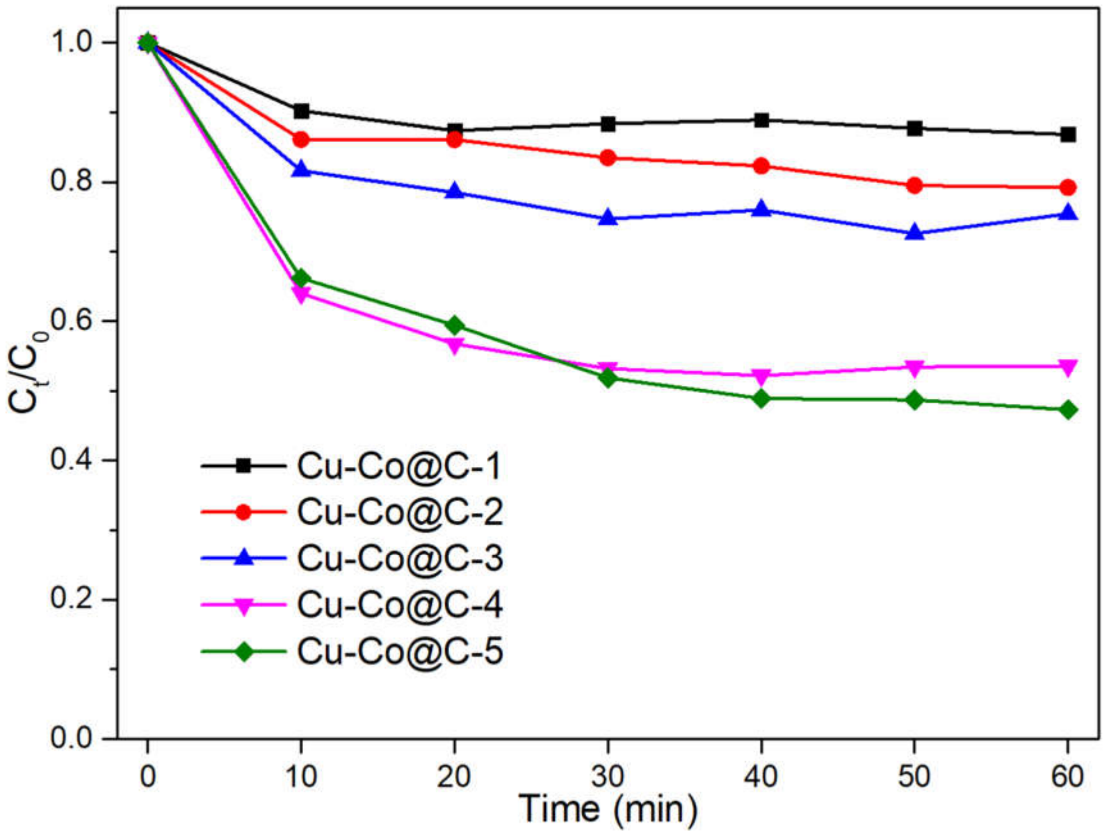

Figure S2. The adsorption of phenol on different materials in the absence of PS. catalyst dosage: 0.5 g L^{-1}, $[Phenol]_0$ = 20 mg L^{-1}.

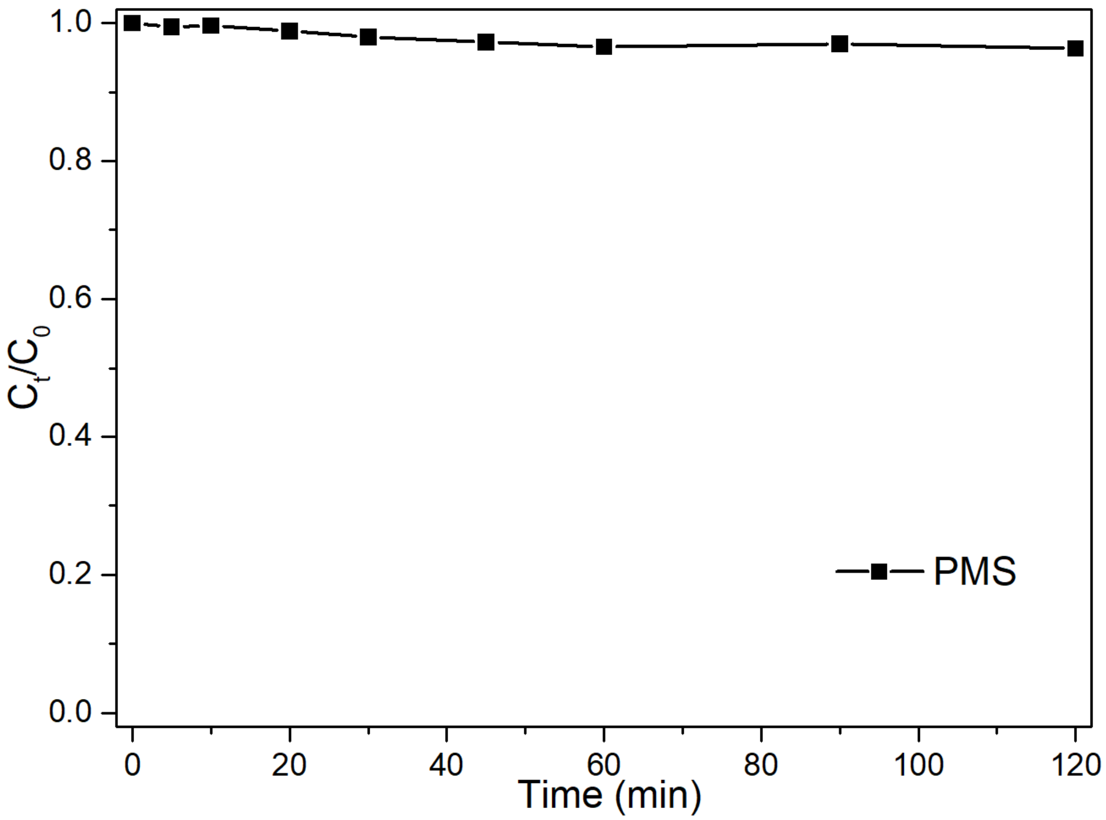

Figure S3. The degradation of phenol by PS without any catalysts. $[Phenol]_0$ = 20 mg L^{-1}, [PMS] = 2 mmol L^{-1}.

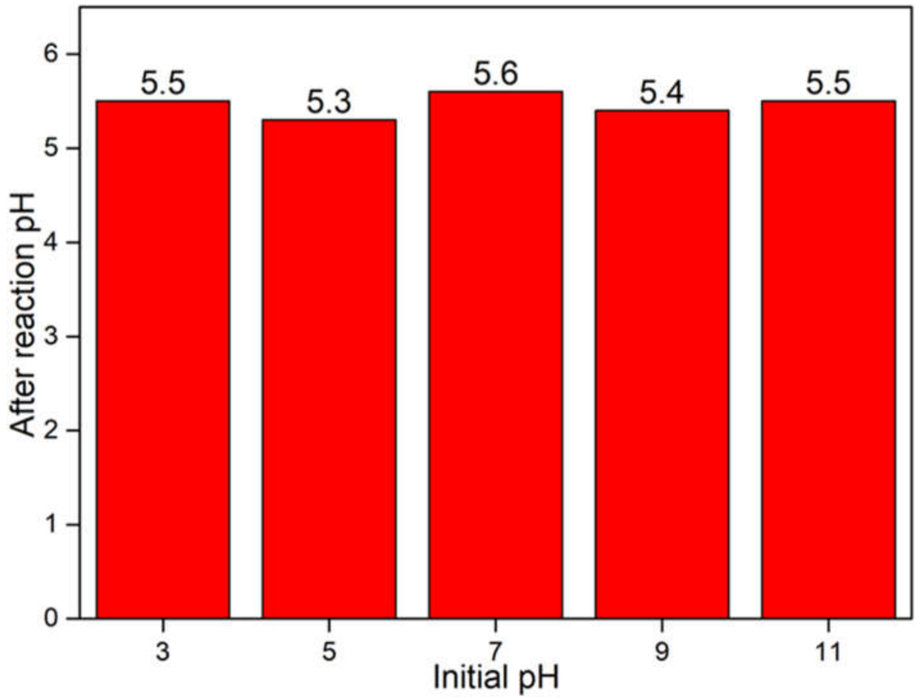

Figure S4. The variation law of various initial pH values after reaction.

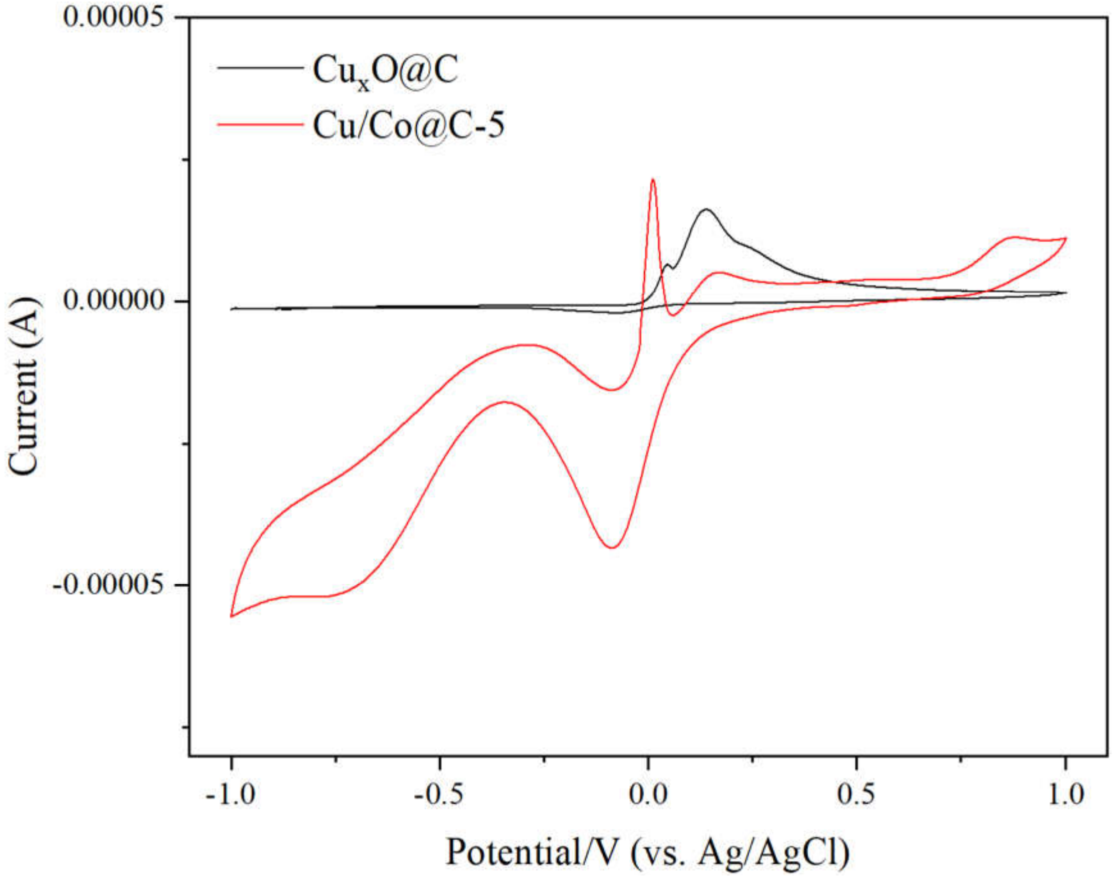

Figure S5. The cyclic voltammetry curves of Cu-Co@C-5 and monometallic (Cu_xO@C).

Table S1. The BET specific surface (S_{BET}), pore volume, and BJH average pore diameter of Cu-Co@C.

Sample	S_{BET} ($m^2\ g^{-1}$)	Pore volume ($cm^{-3}\ g^{-1}$)	BJH Average pore diameter (nm)
Cu-Co@C-1	112.49	0.19	11.59
Cu-Co@C-2	109.05	0.17	13.31
Cu-Co@C-3	121.09	0.13	10.96
Cu-Co@C-4	175.76	0.12	8.69
Cu-Co@C-5	189.45	0.09	7.75

Table S2. The content of elements in Cu-Co@C-5 before and after the reaction.

Element		Before reaction	After reaction
Co (at.%)	Total	7.8	9.7
	Co^0	55.8	19.1
	Co^{2+}	13.2	41.8
	Co^{3+}	31.0	39.2
Cu (at.%)	Total	2.8	0.6
	Cu^0	59.2	40.1
	Cu^+	13.6	20.2
	Cu^{2+}	27.2	39.7
O (at.%)	Total	8.1	23.4
	C=O	69.3	74.5
	C-OH	20.5	19.9
	Me-O	10.2	5.6
C (at.%)	Total	81.3	66.3

Table S3. Comparison of activation PDS/PMS for phenol degradation by different catalyst.

Catalyst	Oxidan	Optimal reaction conditions	Performance	Ref.
CuO	PMS	[phenol]$_0$= 50 mg/L; [CuO]= 1 g/L; [PMS]$_0$= 2 mM; pH 7	65% in 60 min	[1]
Cu-Cu_xO@C	PDS	[PS] = 4 mmol L^{-1}, [activator]= 0.5 g L^{-1}, [PhOH]$_0$ = 20 mg L^{-1}	100% in 105 min	[2]
CuO/Fe_3O_4	PDS	[PDS]$_0$= 5.0 mM, [phenol]$_0$= 0.1 mM, [CuO/Fe3O4] = 0.3 g/L, pH 5.6	80% in 120 min	[3]
CuMgFe-LDO	PDS	[phenol] $_0$= 0.1 mM, [CuMgFe-LDO] = 1.0 g/L,[PDS] $_0$= 0.5 mM, pH 6.4	100% in 30 min	[4]
$LaCo_{0.4}Cu_{0.6}O_3$	PMS	[phenol] $_0$= 20 mg/L, [PMS] $_0$= 0.20 g/L,[catalyst] = 0.1 g/L, pH 7	~100% in 12 min	[5]
CuCo@MnO_2	PMS	[catalyst] = 0.1 g/L, [PMS] $_0$= 0.5 g/L, [phenol] $_0$= 30 mg/L	100% in 100 min	[6]

References

[1] F. Ji, C. Li, L. Deng, Performance of CuO/Oxone system: Heterogeneous catalytic oxidation of phenol at ambient conditions, Chemical Engineering Journal, 178 (2011) 239-243.

[2] X. Li, X. Min, X. Hu, Z. Jiang, C. Li, W. Yang, F. Zhao, In-situ synthesis of highly dispersed Cu-CuxO nanoparticles on porous carbon for the enhanced persulfate activation for phenol degradation, Separation and Purification Technology, 276 (2021).

[3] Y. Lei, C.-S. Chen, Y.-J. Tu, Y.-H. Huang, H. Zhang, Heterogeneous Degradation of Organic Pollutants by Persulfate Activated by CuO-Fe3O4: Mechanism, Stability, and Effects of pH and Bicarbonate Ions, Environmental Science & Technology, 49 (2015) 6838-6845.

[4] Y. Chen, J. Yan, D. Ouyang, L. Qian, L. Han, M. Chen, Heterogeneously catalyzed persulfate by CuMgFe layered double oxide for the degradation of phenol, Applied Catalysis a-General, 538 (2017) 19-26.

[5] S. Lu, G. Wang, S. Chen, H. Yu, F. Ye, X. Quan, Heterogeneous activation of peroxymonosulfate by LaCo1-xCuxO3 perovskites for degradation of organic pollutants, Journal of Hazardous Materials, 353 (2018) 401-409.

[6] A. Khan, Z. Liao, Y. Liu, A. Jawad, J. Ifthikar, Z. Chen, Synergistic degradation of phenols using peroxymonosulfate activated by CuO-Co3O4@MnO2 nanocatalyst, Journal of Hazardous Materials, 329 (2017) 262-271.
